# Mediating Effects of Social Support on Depression and Suicidal Ideation in Older Korean Adults With Hypertension Who Live Alone

**DOI:** 10.1097/jnr.0000000000000292

**Published:** 2019-05-20

**Authors:** Eun Jeong NAM, Jong-Eun LEE

**Affiliations:** 1 MSN, RN, Doctoral Candidate, College of Nursing, The Catholic University of Korea, Seoul, Republic of Korea; 2 PhD, RN, APHN, Associate Professor, College of Nursing, The Catholic University of Korea, Seoul, Republic of Korea.

**Keywords:** older adult, living alone, depression, suicidal ideation, social support

## Abstract

**Background:**

Older adults who live alone are less physically and emotionally healthy and report higher levels of depression relative to those who do not live alone. Suicide is the most problematic health issue reported by older adults who live alone. In particular, vulnerable older adults who live alone experience difficulty obtaining self-care and medical services; therefore, early detection of depression is difficult, and there are few opportunities to implement suicide prevention strategies in this population. In addition, social support for depression is an important factor affecting illness and economic vulnerability in older adults who live alone.

**Purpose:**

This study aimed to examine the relationship between depression and suicidal ideation in vulnerable older Korean adults with hypertension and to explore the mediating effect of social support on this relationship.

**Methods:**

The study used a cross-sectional design. Vulnerable older adults who were 65 years old or older with hypertension and who received home visit services from a public health center were invited to participate. The participants completed structured questionnaires, including the 15-item Short-Form Geriatric Depression Scale, 12-item Multidimensional Scale of Perceived Social Support, and 19-item Scale for Suicidal Ideation, and provided information regarding their demographic characteristics, health status, and economic status. Descriptive and correlation analyses were performed to examine the correlations among these variables. The three-step regression analysis method proposed by Baron and Kenny was used to examine the mediating effect of social support.

**Results:**

The mean depression, social support, and suicidal ideation scores of participants were 23.64 (± 2.04), 35.94 (± 15.40), and 7.80 (± 7.73), respectively. In addition, depression was negatively correlated with social support (*r* = −.27) and positively correlated with suicidal ideation (*r* = .21), whereas social support was negatively correlated with suicidal ideation (*r* = −.35). Social support mediated the relationship between depression and suicidal ideation (*Z* = 2.69).

**Conclusions:**

Social support was identified as an important variable for older adults with chronic illness who lived alone. Interventions that include social support hold the potential to reduce depression and suicidal ideation in this population.

## Introduction

The social burden and prevalence of chronic diseases have increased because of the aging population and lifestyle changes (Cha & Yun, 2015). Chronic diseases were prominent in the 2015 ranking of causes of death for Koreans aged 65 years or older, with heart disease, cerebrovascular disease, and diabetes ranked as the second, third, and fifth most common causes of mortality, respectively. In addition, medical expenses for older adults exhibited an upward trend, accounting for 13.4% of the total medical expenses in 2005 and 36.8% in 2015 (Korean Statistical Information Service, 2016). According to a 2014 survey examining actual conditions affecting the population of older adults in Korea, the overall prevalence rate for chronic diseases was 89.2%, and the proportion of older adults with hypertension (56.7%), which causes cerebrovascular disease, was higher, relative to those for other chronic diseases (Korea Institute for Health and Social Affairs, 2014).

Furthermore, as society has aged, family structure has changed from multifamily households, in which children live with and support their parents, to childless married couples or single-person households. In addition, the proportion of Koreans aged 65 years or older in single-person households was 4.9% in 2005 and 6.4% in 2015, with this percentage expected to reach 11.8% in 2030 if the current trend persists (Korean Statistical Information Service, 2016). Moreover, the household incomes of 53.6% of older adults who live alone are below the minimum cost of living. Therefore, many older adults who live alone experience economic hardship (Jeong, 2015). Moreover, the prevalence of chronic diseases in older adults who live alone is 93.2%, which is higher relative to that of older adults who live with their children (89.6%) and spouses (87.1%; Korea Institute for Health and Social Affairs, 2014). Moreover, according to a 2014 survey examining actual conditions affecting the elderly population in Korea, which was conducted by a researcher at the Korea Institute for Health and Social Affairs, the most serious problems experienced by older adults were related mainly to health, psychological, and economic issues (e.g., the absence of caregivers during illness [37.2%], psychological anxiety and loneliness [24.4%], and economic anxiety [21.6%]), with 43.7% of respondents describing their health conditions as serious (Korea Institute for Health and Social Affairs, 2014). In addition, older adults who lived alone reported multiple health problems and exhibited poorer physical and emotional health (You & Park, 2003) and higher levels of depression relative those who did not live alone (Baek & Lee, 2014).

The suicide rate for older adults aged 65 years or older in Korea from 2012 to 2015 was 54.8 in 100,000 persons, which is higher than the average of 18.4 in 100,000 persons reported by member countries of the Organization for Economic Cooperation and Development during the same period. Moreover, Korea has had the highest elderly suicide rate of any Organization for Economic Cooperation and Development country every year since 2003 (Korean Statistical Information Service, 2016). The suicide rate among elderly Koreans having chronic diseases is significantly higher than among those without chronic diseases (Song, Son, & Park, 2010) and even higher among those in this subgroup who live alone (Sohn, 2012). The long-term burdens of managing incurable chronic diseases (Sohn, 2012), difficulties encountered in living with chronic diseases (Oh, Bae, & Kim, 2006), and limited range of activities and persistent pain caused by chronic diseases (Jang et al., 2014) all likely contribute to the high suicide rate among the older adults with chronic diseases. A systematic literature review of suicidal ideation among the older adults who live alone in Korea suggested that the variables affecting suicidal ideation are depression (among the individual system factors), family structure or living alone (among the family system factors), and social severance and lack of social support (among the community factors; Song et al., 2010). In another study, vulnerable older adults who lived alone and had chronic diseases experienced mental depressive symptoms and economic difficulties (Jeong, 2015). In addition, the prevalence rate of depressive symptoms among older Korean adults who live alone is 43.7%, which is higher than the 33.1% observed for the overall older adult population (Korea Institute for Health and Social Affairs, 2014). In particular, the use of positive strategies to cope with health problems, including hypertension and depression, may help people lead healthier lives in old age, whereas coping negatively or failing to respond appropriately to problems may lead to the extreme decision to commit suicide (S. H. Kim & Choi, 2007). Unlike in other age groups, suicide in old age is closely associated with negative experiences such as illness, solitude, alienation, and loss, as these events occur frequently in old age and may influence older adults to perceive suicide as a means of avoiding pain in life (Kwon, Um, & Kim, 2012). In particular, a study on older adults who lived alone found depression to be the factor with the greatest effect on suicidal ideation (Sohn, 2012). The physical and social factors leading to aging, illness, and economic difficulties increase vulnerability to depression in older adults (Alexopoulos, 2005). Notably, older adults who live alone are even more vulnerable to these factors and thus more vulnerable to depression (Sohn, 2012). G. Y. Lee and Choi in 2001 found that depression directly affected suicidal ideation in older adults who lived alone (G. Y. Lee & Choi, 2013).

Social support has been identified in several previous studies as a mediating factor of depression (Choi & Won, 2012; Greenglass, Fiksenbaum, & Eaton, 2006; Han, Song, Song, & Kim, 2013). In addition, depression levels in older adults who live alone have been shown to be higher relative to those observed in older adults who do not live alone because of a lack of social networks and support systems (Choi & Won, 2012). In a study involving Korean-American individuals, those who formed relationships with and received support from relatives or neighbors were more likely to exhibit good mental health relative to those without these relationships (S. H. Kim & Choi, 2007). Furthermore, a study involving 4,489 older adults who lived in Singapore showed that the depression scores of older adults who lived alone were higher relative to those of older adults who did not live alone, regardless of gender (Han et al., 2013). Levels of social support for older adults who live alone are lower relative to those for older adults who do not live alone, resulting in social isolation, which may ultimately lead to suicide (Manthorpe & Iliffe, 2010). Moreover, if the sense of isolation experienced by older adults who live alone and do not have a strong social network remains unresolved, this sense may persist and ultimately lead to suicide (E. J. Lee, Bae, & Um, 2010). In addition, levels of education and income in vulnerable older adults who live alone are likely to be lower relative to those observed in older adults who do not live alone, with these factors potentially further exacerbating social isolation and ultimately leading to suicide (Y. J. Kim, 2009).

In addition, many older adults with chronic diseases, including hypertension, choose to discontinue treatment as they experience various physical side effects and psychological changes such as depression, leading to dysfunction, cognitive decline, and difficulties in daily life (Oh et al., 2006).

Access to medical services is typically lower for older adults who live alone than for older adults who do not live alone, with lack of related information a particularly serious problem. Poor access to medical services has been shown to result in low levels of prevention and early treatment (Sohn, 2012). In addition, this situation makes self-care difficult and necessitates active mediation. Moreover, vulnerable older adults with hypertension who live alone are considered at a high risk of suicide because they experience physical difficulties and social isolation simultaneously. Further research on this population is needed.

Few studies have examined the relationship between suicide attempts and depression, and the social support that mediates these two variables in older adults with chronic illness who live alone. Most studies have either worked to identify factors affecting suicide, depression, and social support in older adults (Jang et al., 2014; Kang, Kim, Lee, Jung, & Ma, 2012) or compared levels of suicidal ideation and depression between older adults who live alone and those who live with others (Y. J. Kim, 2009). As mentioned above, older adults who live alone and have hypertension are very physically and mentally vulnerable and at a higher risk of suicidal ideation than their peers. Thus, older adults with hypertension were selected as the subjects of this study and were examined to elicit the relationship between depression and social support, which are important factors of suicidal ideation. This study is expected to serve as a basis for developing interventions to reduce suicidal ideation among the older adults with hypertension who live alone and, by extension, to encourage older adults who live alone and have chronic diseases to seek out and participate in these interventions.

As mentioned above, depression could be significantly associated with social support and suicidal ideation in older adults who live alone, particularly those with hypertension, which is the most common chronic disease in this population; however, no studies have been conducted to examine this issue.

This study examined the mediating effect of social support on the relationship between depression and suicidal ideation in vulnerable older adults with hypertension who live alone. The objectives of this study were the following:

1. Examine the demographic characteristics, depression, social support, and suicidal ideation of vulnerable older adults with hypertension who live alone.
2. Examine the relationships among depression, social support, and suicidal ideation in vulnerable older adults with hypertension who live alone.
3. Determine the mediating effects of social support on the relationship between depression and suicidal ideation in vulnerable older adults with hypertension who live alone.

## Methods

### Study Design

A cross-sectional study was conducted to analyze the mediating effects of social support on the relationship between depression and suicidal ideation in vulnerable older adults with hypertension living alone in Korea.

### Participants

Older adults who lived alone and who either received home visitation services provided by a public health center (i.e., Medical Benefits 1 and Medical Benefits 2) or belonged to a low-level health insurance group were recruited from among registered users of a public health center, the On-site Health Care Project, in a district in Seoul, South Korea. The inclusion criteria for the study were as follows: (a) aged 65 years or older, (b) diagnosed with hypertension by a physician or currently using hypertension medicine, and (c) able to understand the purpose of the study and to communicate. The exclusion criteria were having (a) an acute disease (e.g., myocardial infarction), (b) terminal cancer, or (c) cognitive impairment.

In the multiple regression analysis, a minimum of 119 participants was calculated using G*Power 3.1 (Heinrich Heine University, Dusseldorf, Germany; Faul, Erdfelder, Buchner, & Lang, 2009), with a significance level .05, a power of 0.95, a moderate effect size of .15, and three independent variables (depression, social support, and suicidal ideation). Data were collected from 215 potential participants; 53 were excluded because of incomplete responses. Thus, data from 162 participants were included in the analysis.

### Measures

#### Depression

The Geriatric Depression Scale Short Form, Korean Version (GDSSF-K; Kee, 1996), which consists of 15 items from the original 30-item Geriatric Depression Scale (Yesavage et al., 1982) and was standardized for use with Korean populations, was used to measure depression. Responses for the 15 items are provided on a dichotomous scale (yes/no), with total possible scores ranging from 15 to 30 and higher scores indicating more severe depression symptoms. Cronbach's αs were .95 for the original scale in Yesavage et al.'s study, .88 for the GDSSF-K in Kee's study, and .93 for the GDSSF-K in this study. The scale was used in a previous study on a population of elderly Koreans living alone, which supported the scale's construct validity (Sohn, 2012).

#### Social support

The Multidimensional Scale of Perceived Social Support (Zimet, Dahlem, Zimet, & Farley, 1988) was used to measure social support. This scale consists of 12 items divided equally among the following three categories: neighbors (e.g., “I have people around me when I need them”), family (“My family helps me willingly”), and friends (“My friends help me willingly”). Responses are provided using a 7-point Likert scale, ranging from 1 = *not at all* to 7 = *very much*. The range of possible total scores for each category is 4–28, and the range of possible total scale scores is 12–84, with higher scores indicating stronger social support. Cronbach's αs for the scale were .84 in Zimet et al.'s study and .93 in this study. The construct validity of the Korean version of the Multidimensional Scale of Perceived Social Support was tested previously on a population of Korean older adults (Kang et al., 2012).

#### Suicidal ideation

The Korean version (Shin, Park, Oh, & Kim, 1990) of the Scale for Suicide Ideation (Beck, Kovacs, & Weissman, 1979) was used to measure suicidal ideation in this study. The scale consists of 19 items pertaining to impulses associated with suicide that were experienced during the preceding year. Responses are provided on a 3-point scale ranging from 0 = *not at all* to 2 = *a lot*, and the range of potential total scores is 0–38, with higher scores indicating stronger suicidal ideation. Cronbach's αs were .92 for the original scale in Beck et al.'s study, .87 for the Korean version of the scale in Shin et al.'s study, and .93 for the Korean version of the scale in this study. The use of this scale in a previous study on an elderly Korean population living alone supported its construct validity (Sohn, 2012).

### Procedures

Ethical approval (MC12QISI0060) for this study was granted by the institutional review board at the institution with which the authors were affiliated. Thirteen home visiting nurses employed by the public health center collected the data between July and August 2012. The researchers provided a data collection training session for the nurses, who all had backgrounds in data research. The nurses visited participants' homes and explained the study purpose and procedures and the voluntary nature of participation. In addition, they informed the participants that they could withdraw from the study at any time without adverse consequences. All of the participants provided informed consent. The home visiting nurses read the questionnaire to the participants and recorded their responses, which took approximately 40 minutes in total.

### Data Analysis

Data were analyzed using IBM SPSS Statistics Version 18.0 (IBM, Armonk, NY, USA). Participants' demographic characteristics and depression, social support, and suicidal ideation scores were analyzed using descriptive statistics. Proportions and frequencies were calculated for participants' demographic characteristics, and means and standard deviations were calculated for participants' depression, social support, and suicidal ideation scores. Pearson's correlation analysis was performed to analyze the correlations between depression, social support, and suicidal ideation.

The mediating effect of social support on the relationship between depression and suicidal ideation was calculated using the three-step regression method proposed by Baron and Kenny in 1986. The effect of depression on social support was examined in Step 1; the effect of depression on suicidal ideation was examined in Step 2; and, in Step 3, the effect of depression on suicidal ideation was examined to determine whether it was weaker relative to that observed in Step 2. After the mediating effect of social support was shown in the regression analysis, a Sobel test (Sobel, 1982) was performed to determine statistical significance.

## Results

### Demographic Characteristics of Participants

The mean age of participants was 75.75 (± 5.85) years, and 24.7% and 75.3% were male and female, respectively. Most (93.2%) were unemployed or had retired from work and were receiving government subsidies (90.7%) to meet their living expenses. With respect to educational level, 30.3% reported having no formal education (Table 1). On average, participants had lived alone for 21.33 (± 17.94) years and had 1.75 (± 1.60) children. With respect to marital status, the proportion of widowed participants (59.9%) was higher relative to those for other types of marital status. In addition, 27.2% and 25.3% of the participants reported that they lived alone because, respectively, they had no family and their children had married. Regarding the greatest difficulty faced by participants because of living alone, “economic difficulties” was reported most frequently (43.2%), followed by “poor health” (25.9%) and “loneliness” (17.3%).

**TABLE 1.**
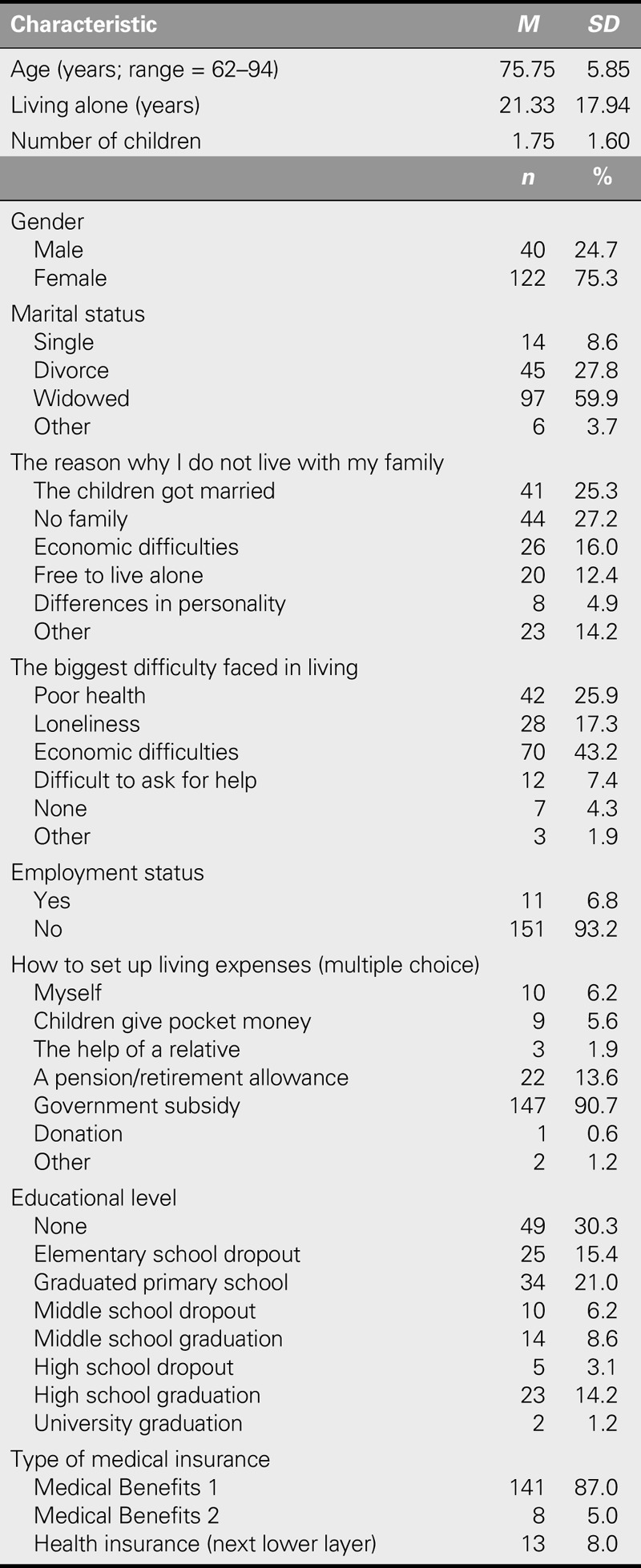
General Characteristics of the Participants (*N* = 162)

### Depression, Social Support, and Suicidal Ideation Scores

The mean depression score of participants was 23.64 (± 2.04), which indicated severe depression. In addition, their mean social support and suicidal ideation scores were 35.94 (± 15.40) and 7.80 (± 7.73), respectively. Regarding sources of social support, the mean score for neighbors was the highest (13.29 ± 6.37), followed by those for family (11.79 ± 6.24) and friends (10.86 ± 5.84; Table 2).

**TABLE 2.**
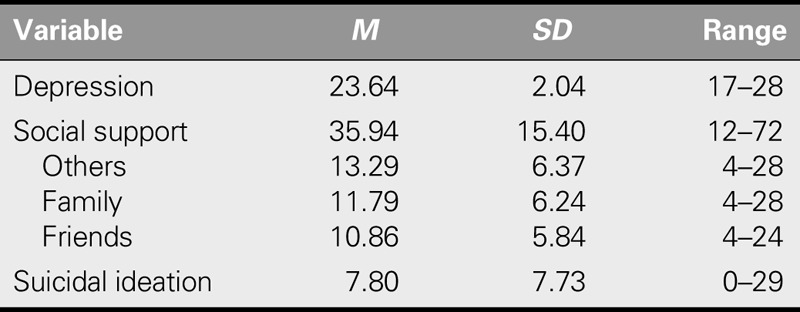
The Level of Depression, Social Support, and Suicidal Ideation (*N* = 162)

### Correlation Analysis

Results of the correlation analysis showed that depression was negatively correlated with social support (*r* = −. 27, *p* = .001) and positively correlated with suicidal ideation (*r* = .21, *p* = .007) and that social support was negatively correlated with suicidal ideation (*r* = −.35, *p* < .001; Table 3).

**TABLE 3.**
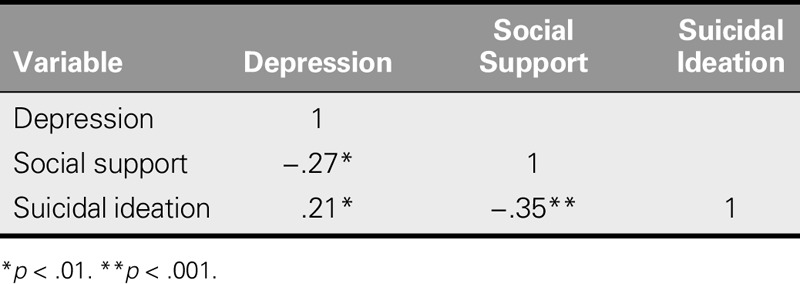
Correlations Between Variables (*N* = 162)

### The Mediating Effect of Social Support

The results of the three-step regression analysis (Baron & Kenny, 1986) are shown in Table 4. The results regarding the assumptions of this analysis were as follows: The Durbin–Watson index was 1.87, indicating no autocorrelation of residuals, and the variance inflation factor was 1.08, indicating no multicollinearity (score < 10).

**TABLE 4.**
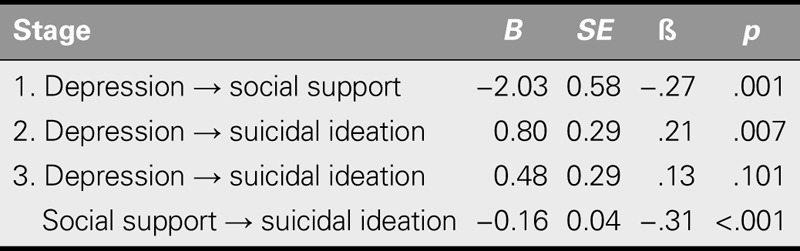
Parametric Analysis Method by Baron and Kenny: Three Stages (*N* = 162)

**Figure 1.**
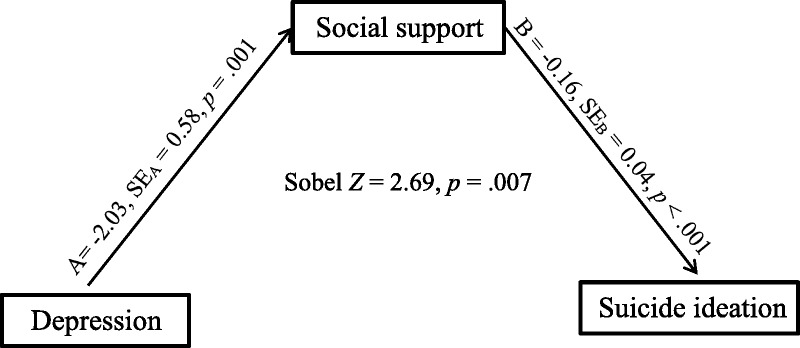
Mediating model.

The results of Step 1 of the regression analysis showed a significantly negative correlation between depression and social support (β = −.27, *p* = .001). The results of Step 2 of the regression analysis showed a significantly positive correlation between depression and suicidal ideation (β = .21, *p* = .007). The results of Step 3 of the regression analysis showed that social support correlated negatively with suicidal ideation (β = −.31, *p* < .001) and that depression was not correlated with suicidal ideation (β = .13, *p* = .101; Table 4). Therefore, social support was supported as a mediator between depression and suicidal ideation (*Z* = 2.69, *p* = .007; Figure 1).

## Discussion

This study examined the mediating effect of social support on the relationship between depression and suicidal ideation in vulnerable older adults with hypertension who lived alone. The mean depression score of participants indicated that they experienced severe depression. This finding is consistent with those of a previous study in which depression scores in older adults who lived alone were significantly higher relative to those of older adults who did not live alone and even higher in participants with chronic diseases (Sohn, 2012). In addition, another study found that the various physical, psychological, and social factors associated with aging, illness, economic difficulties, and social isolation increased the vulnerability of elderly individuals to depression (Alexopoulos, 2005). The results of other studies dovetail with the severe depression that was observed in this study in the subgroup of economically vulnerable participants.

The mean social support score for the participants in this study was lower relative to that in a previous study of community-dwelling older adults who were not living alone (Kang et al., 2012). Moreover, the participants in this study were vulnerable and experienced illness and economic hardship, and their mean social support score was lower relative to that observed for healthy older adults who did not live alone. According to a survey of 10,451 Korean older adults (Korea Institute for Health and Social Affairs, 2014), only 28.9% were gainfully employed and 20.0% were receiving government subsidies. Participants in this study had a comparatively lower number of children, a higher unemployment rate, and a higher ratio of government-subsidy recipients. These findings support that living with family members provides emotional support for older adults via social contact (Sohn, 2012).

The mean suicidal ideation score of participants in this study was considerably higher than that observed in Sohn's (2012) study of older adults who lived alone. However, 40.0% of those in Sohn's study were employed and 37.4% received social contact more than once per week, whereas 93.2% of the participants in this study were not employed and thus had few opportunities for social contact.

Furthermore, a negative correlation was found between depression and social support. This finding is consistent with those of a previous study in which older adults who lived alone and had weak social networks and social support systems exhibited high levels of depression (Choi & Won, 2012; Han et al., 2013; S. H. Kim & Choi, 2007). Moreover, the findings of this study are also consistent with those of a study conducted by Greenglass et al., which showed that social support in old age is related to the effects of depression.

Furthermore, a positive correlation was found between depression and suicidal ideation, indicating that higher levels of depression led to higher levels of suicidal ideation in older adults with hypertension who lived alone. This is consistent with previous studies that found depression to be the most prominent cause of suicide among older individuals (Kwon et al., 2012) and that older adults who lived alone failed to cope actively with depression or to identify appropriate means to ameliorate their depressive symptoms and thus considered suicide to avoid their problems (Sohn, 2012).

The results also found a negative correlation between social support and suicidal ideation. This finding is consistent with the findings of a previous study in which older adults living alone received insufficient social support, causing social isolation, which increased levels of suicidal ideation (Manthorpe & Iliffe, 2010). The findings of this study are also consistent with those of Y. J. Kim, who found that older adults who lived alone were more likely to commit suicide than their peers who lived with others and that lower levels of education and income increased vulnerability and led to strong feelings of loneliness (Y. J. Kim, 2009). Furthermore, the findings of this study are consistent with the results of a study that emphasized the importance of improving support systems for older adults who live alone and receive poor social support, as their circumstances significantly increase their risk of attempting suicide (E. J. Lee et al., 2010).

The results of this study indicate that social support mediates the relationship between depression and suicidal ideation in vulnerable older adults with hypertension. This suggests that social support is a significant variable that explains and mediates the effect of depression on suicidal ideation. In other words, depression in this population exerts an indirect influence via social support on suicidal ideation. Moreover, a study conducted by Greenglass et al. indicated that social support exerted a mediating effect on depression in the population of older adults. In G. Y. Lee and Choi's study, depression was shown to exert a direct effect on suicidal ideation in older adults who lived alone and social support was shown to exert an indirect effect on suicidal ideation via the pathway between social isolation and depression, which is inconsistent with the results of this study. This discrepancy between the findings may be related to the inclusion of healthy older adults by G. Y. Lee and Choi in their sample and the inclusion in this study of older adults with hypertension, which is a chronic illness that is a risk factor for social isolation and vulnerability. Thus, social support may have been of greater importance to the participants in this study than in G. Y. Lee and Choi's study.

In addition, economically and socially vulnerable older adults who live alone may experience difficulty accessing medical services in a timely manner. Therefore, health problems such as depression should be considered a particularly serious issue in this population (Choi & Won, 2012). On the basis of the results of a study conducted by Heaney and Israel (2008), which indicated that well-established social support systems reduced negative emotion, including loneliness and depression; increased access to healthcare information; and ultimately reduced the risk of illness, the promotion and establishment of a social support system is of utmost importance in improving healthcare for vulnerable older adults with hypertension who live alone. Furthermore, the results of this study showed that, of the social support resources considered, social support received from neighbors was more influential than that received from family and friends. This indicates that securing support resources from individuals on the periphery of the social lives of older adults with hypertension who live alone is important when establishing a social support system for this population. Therefore, to maintain adequate levels of social health in vulnerable older adults with diseases such as hypertension who live alone, interventions that measure depressive symptoms and establish and maintain social support systems that consist of individuals on the periphery of patients' social lives are necessary. Moreover, support resources consisting of individuals on the periphery of patients' lives should provide practical support in active interventions that promote and enhance healthcare for vulnerable older adults who live alone and others in the same age bracket through direct contact between volunteers and participants.

The participants in this study were vulnerable older adults with hypertension who lived alone in a metropolitan area, received home-visiting nursing services from public health centers, and belonged to low-level health insurance groups. Therefore, the generalizability of the results to older adults with different health conditions is limited. Furthermore, as this study used a cross-sectional and correlational design, no inferences are able to be made regarding the causal relationships between the variables.

Despite these limitations, social support was shown to be vital to the emotional well-being of vulnerable older adults with chronic diseases who live alone. Future interventions should focus greater attention on social support and improve supportive social systems for older adults who live alone.

### Conclusions

The results of this study suggest that social support mediates the effect of depression on suicidal ideation, indicating that depression, although not having a direct effect on suicidal ideation, has an indirect effect via social support. In addition, social support was identified as a crucially important factor in the lives of older adults who lived alone, who belonged to vulnerable groups, and who had chronic diseases. Therefore, interventions that include social support hold the potential to reduce depression and suicidal ideation in the vulnerable population of older individuals who live alone.
